# Emerging Functional Polymer Composites for Tactile Sensing

**DOI:** 10.3390/ma16124310

**Published:** 2023-06-11

**Authors:** Jia-Jin Lian, Wen-Tao Guo, Qi-Jun Sun

**Affiliations:** School of Physics and Optoelectronic Engineering, Guangdong University of Technology, Guangzhou 510006, China

**Keywords:** tactile sensor, functional polymer composites, electronic skin

## Abstract

In recent years, extensive research has been conducted on the development of high-performance flexible tactile sensors, pursuing the next generation of highly intelligent electronics with diverse potential applications in self-powered wearable sensors, human–machine interactions, electronic skin, and soft robotics. Among the most promising materials that have emerged in this context are functional polymer composites (FPCs), which exhibit exceptional mechanical and electrical properties, enabling them to be excellent candidates for tactile sensors. Herein, this review provides a comprehensive overview of recent advances in FPCs-based tactile sensors, including the fundamental principle, the necessary property parameter, the unique device structure, and the fabrication process of different types of tactile sensors. Examples of FPCs are elaborated with a focus on miniaturization, self-healing, self-cleaning, integration, biodegradation, and neural control. Furthermore, the applications of FPC-based tactile sensors in tactile perception, human–machine interaction, and healthcare are further described. Finally, the existing limitations and technical challenges for FPCs-based tactile sensors are briefly discussed, offering potential avenues for the development of electronic products.

## 1. Introduction

Tactile sensing, encompassing the discernment and quantification of physical attributes including force, pressure, texture, shape, and temperature through direct contact with objects or surfaces, constitutes a fundamental cognitive ability inherent in both human and animal organisms. The human skin is equipped with various sensory receptors, including pain receptors, cold receptors, warm receptors, and mechanoreceptors that specialize in detecting different types of mechanical stimuli. Through these receptors, tactile sensing provides us with perceptible insights into the properties of materials and surfaces, particularly in terms of contact force and thermal characteristics. This wealth of tactile information not only aids our survival but also enables us to explore and respond to the external world in a more nuanced manner. This extraordinary capacity underpins a vast array of tasks, spanning from intricate manipulation and exploratory undertakings to sophisticated modes of communication. Moreover, tactile sensing extends profound relevance within the domain of artificial systems such as robots, prosthetics, and smart textiles [1,2,3]. By imbuing these systems with the capability to perceive and interpret tactile cues, their overall performance, functional versatility, and interactive potential can be significantly enhanced, propelling them to the forefront of technological innovation and human–machine interaction. Various types of tactile sensors have been developed to achieve better tactile sensing based on different materials and mechanisms. Among the various mechanisms employed, resistive, capacitive, piezoelectric, triboelectric, optical, and magnetic sensing principles have been extensively utilized. Each mechanism offers unique advantages and is suitable for specific applications.

However, conventional tactile sensors based on rigid materials or complex structures have limitations in terms of flexibility, sensitivity, durability, and cost. Therefore, there is a need for developing new materials and designs for tactile sensing that can overcome these challenges and enable novel functionalities. With the rapid advancements in materials science and engineering, functional polymer composites (FPCs) have emerged as promising candidates for developing flexible and sensitive tactile sensors. These composites combine the unique properties of polymers with functional additives, enabling them to sense and transduce external stimuli such as pressure, temperature, and deformation into electrical signals [1,4]. FPCs play a key role in tactile sensors, not only affecting the performance and stability of sensors, but also determining the shape and size of sensors.

In recent years, there has been significant progress in the development of FPCs for tactile sensing. These composites exhibit desirable characteristics such as flexibility, stretchability, biocompatibility, and ease of fabrication, making them ideal for applications requiring conformability and sensitivity. Moreover, the integration of functional additives such as conductive fillers, piezoelectric materials, and stimuli-responsive polymers further enhances the sensing capabilities of these composites. The realm of tactile sensing has witnessed remarkable advancements with the emergence of ultrathin conducting or semiconducting materials. Among these materials, notable contenders such as MXene [2], single-crystal silicon nanomembranes [5,6], conjugated polymer-based nanocomposites [7], graphene [8], and molybdenum-disulfide (MoS_2_) [9,10,11] have garnered substantial attention for their potential in the development of tactile sensors. 

This review will highlight the latest advancements in FPCs for tactile sensing. It covers the basic working mechanism, the key properties and requirements for tactile sensors, and several examples of FPCs. The subsequent sections will delve into specific categories of different FPSs-based tactile sensors, including the combination of new materials, unique structure designs, fabrication techniques, and the performance of these composites in detecting and transducing tactile stimuli. The practical applications of FPCs in areas such as electronic skin (e-skin), human–machine interfaces, and healthcare, associated with the challenges and limitations of FPCs will also be discussed. It will identify the key issues that need to be addressed, such as durability, sensitivity, and scalability, to accelerate the translation of these materials from the laboratory to practical applications. By comprehensively analyzing the recent literature, this review aims to offer valuable insights into the development and potential opportunities of FPCs for future research in tactile sensing.

## 2. Mechanisms of Tactile Sensing

**Figure 1:**
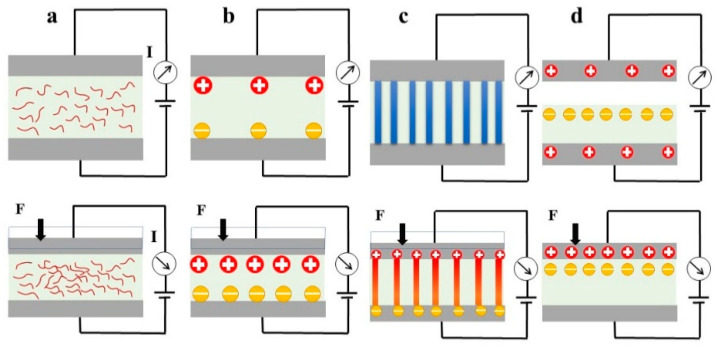
Schematics of typical transduction mechanisms: (**a**) Piezoresistive. (**b**) Capacitive. (**c**) Piezoelectric. (**d**) Triboelectric.

The fundamental mechanism of tactile sensors involves converting the measured signals into physical signals (e.g., voltage, current, capacitance, and resistance), which can be achieved through two basic domains: nonelectrical field coupling and electrical field coupling [3]. Advances in fabrication techniques have enabled the integration of various transduction mechanisms into tactile sensors, including capacitive, piezoresistive, piezoelectric, triboelectric, optical, magnetic-force-based methods, and so on. This section focuses on the distinct types of transduction mechanisms: piezoresistive, capacitive, piezoelectric, and triboelectric (Figure 1).

### 2.1. Piezoresistive Tactile Sensing

The principle of piezoresistive sensors is the exertion of mechanical force upon the sensor’s surface elicits a change in its resistance characteristics, consequently giving rise to the generation of an electrical signal, exhibited in Figure 1a. This change is caused by two effects: the geometric effect, which changes the length and cross-sectional area of the material, and the piezoresistive effect, which changes the resistivity of the material [12]. The active material’s resistance, denoted as R, can be represented by the following equation, R=ρ×L /A, where ρ, *L*, and *A* represent resistivity, total length of the sensitive element, and the cross-sectional area of the sensitive element, respectively. The geometric effect is the change in length L and cross-sectional area A due to strain. A for a strain sensor is determined by the interplay of geometry and resistivity. The variation in resistance can be mathematically described as ∆R/R=∆ρ/ρ+(1+2v)ε, where *v* is Poisson’s ratio and ε is strain [13]. The piezoresistive effect is characterized by the material’s resistance changing proportionally to the applied pressure [14].

The piezoresistive effect is more dominant for some materials, such as conductive rubber, ink, silicone, or foam. These materials can be used to make thin-film or layer sensors that generate electrical signals when pressed. However, these sensors are sensitive to temperature changes that affect their resistance and accuracy. To overcome this problem, researchers have used flexible polymer materials with conductive fillers, such as carbon nanotubes, nanoparticles, or fibers [15,16,17]. These materials can improve the conductivity and deformation of the sensor and make it more stable and reliable. Nonetheless, these sensors also have drawbacks such as low frequency response and high hysteresis due to the interactions between the conductive fillers and the polymer matrix or substrate [18].

### 2.2. Capacitive Tactile Sensing

By detecting changes in capacitance, the sensors measure variations in touch, pressure, or other force to estimate the corresponding changes in capacitance, which refers to the capacity of an object to accumulate electric charge (Figure 1b). The main advantages of capacitive sensing over other detection approaches are that it can sense different kinds of materials (skin, plastic, metal, and liquid), and the possibility to achieve long-range sensing with small sensor sizes. Capacitive tactile sensors consist of two conductive surfaces separated by an elastic dielectric layer. The two surfaces are insulated from each other by a material such as air or a polymer. The capacitance of a parallel plate capacitor can be calculated as: $C\;=A\varepsilon_{0}\varepsilon_{r}/d$, where C is capacitance and A is the overlapping area of the two electrodes in square meters; $\varepsilon_{r},\varepsilon_{0}$ represent the permittivity of vacuum space and the dielectric material layer, respectively; and d is the distance between two plates. 

Flexible electrodes can be fabricated by using composites that contain conducting materials. Moreover, microstructures can be designed on the dielectric layer to increase the contact area and deformation range between the dielectric and conductive layers, leading to improved capacitance changes. The traditional way of designing microstructures is adopting microstructures such as pyramids [19], rectangular [20], domes [21], bionic structures [22], hierarchical sea urchin TiO_2_ particle-in-micropore [23], and the interlocked microstructures [24]. They have a larger surface area, which allows for more efficient coupling with the conductive electrodes, leading to improved capacitance changes. The greater contact area between the dielectric and conductive layers enhances the sensitivity and resolution of the capacitive tactile sensor. By incorporating reinforcing elements or designing hierarchical structures, the dielectric material can withstand repeated deformations and maintain its sensing capabilities over an extended period. This durability ensures the longevity and reliability of the capacitive tactile sensor. Some natural materials such as *Epipremnum aureum* leaf [25], rose petals [22], lotus leaves [26], and orange peel [27] can also be used as eco-friendly, readily available, and cost-effective dielectric materials with unique features such as self-cleaning ability and water repellency.

### 2.3. Piezoelectric Tactile Sensing

Utilizing the principles of the piezoelectric effect, piezoelectric tactile sensors operate through the conversion of mechanical energy into electrical energy. When an external force is applied to a piezoelectric material, it generates an electric charge that is directly proportional to the magnitude of the force, as depicted in Figure 1c. In the specific case of ZnO, which is a crystal without a center of symmetry, the arrangement of Zn^2+^ cations and O^2−^ anions is tetrahedral [28]. In the absence of any strain, the charges within the crystal are balanced, and the positive and negative charge centers coincide, resulting in a net dipole moment of zero. However, when a metal plate applies mechanical stress or force, it causes the charge in the crystal to lose equilibrium and generate a dipole moment. The sum of all dipole moments produces a reduction in the macroscopic potential in the direction of the crystal strain, which is known as the piezoelectric potential. 

Another crucial factor in piezoelectric charge is the piezoelectric constant $d_{ij}$, which represents the correlation between the applied mechanical stress and the electric charge generated. The first subscript “i” indicates the direction of the poled dipoles, while the second subscript “j” signifies the applied force. In polycrystalline materials, there are five piezoelectric constants, and their magnitude relationship can be represented as: $d_{15}=d_{24}>d_{33}>d_{31}=d_{32}$ [29].

### 2.4. Triboelectric Tactile Sensing

Utilizing the synergistic interplay between triboelectrification and displacement current, triboelectric tactile sensors have demonstrated the capability to discern and quantify mechanical stimuli, including pressure and vibration. This intriguing phenomenon arises from the interfacial interaction between two distinct materials, leading to the generation of an electric charge through the intricate transfer of electrons (Figure 1d). Triboelectric nanogenerators (TENG), an auspicious avenue for self-powered sensing, inherently possess remarkable flexibility and hold significant promise in terms of high stretchability [30,31]. These innovative devices effectively harness and convert ambient mechanical energy into electrical power, offering an efficient means of energy conversion.

Triboelectric tactile sensors commonly employ a fabrication process that includes depositing a thin layer of triboelectric material onto a conductive substrate. Materials such as polydimethylsiloxane (PDMS) [32], polytetrafluoroethylene (PTFE) [33], polyvinylidene fluoride (PVDF) [34,35], or polyurethane (PU) [34,36] are frequently used as triboelectric materials. The deposition is typically performed onto a conductive substrate, which is commonly a metal plate. Triboelectric tactile sensors offer notable advantages in terms of high sensitivity, fine resolution, and low power consumption. However, their performance can be hindered by their inherent vulnerability to environmental noise, which can considerably impair the signal-to-noise ratio. Consequently, this limitation imposes constraints on the sensors’ overall sensitivity and accuracy. 

## 3. Basic Characteristics for Tactile Sensing

Various functional polymer composites can be used for tactile sensing, depending on the desired properties and applications, such as conductive polymer [37] and mechanoluminescent materials [38]. The fundamental characteristics of tactile sensors are primarily determined by their active layer and substrate materials. The active layer, which is the most critical component of tactile sensors, can sense physical quantities from the external environment including pressure, temperature, and deformation, and convert them into various electronic signals (e.g., current, voltage, resistance, and capacitance). When striving for high-quality soft tactile sensors, careful consideration must be given to the mechanical, chemical, and thermal properties of polymer materials. A competent tactile sensor necessitates high sensitivity, rapid response, robustness, repeatability, and resilience. To cater to the varied needs of sensors under different circumstances, a judicious selection of sensor materials becomes indispensable. Thus, the critical parameters are discussed here.

The sensitivity of the tactile sensor is the ability to respond to an external stimulus signal, usually manifested as the ratio between the change in output signal and the change in stimulus signal. In general, tactile sensitivity is defined as S=dX/dP, where *S* is the sensitivity, *X* is the quantitative output signals, and *P* is the imposed stimuli. It is worth mentioning that the sensitivity is also described as the gauge factor (GF). In terms of piezoresistive tactile sensors, the GF is defined as the ratio of the change in resistance to the original resistance of the strain gauge due to the applied strain. The GF of a capacitive tactile sensor is defined as the ratio of the relative change in electrical resistance to the relative change in length [39]. 

The detection limit refers to the minimum level of force or pressure that the sensor can reliably detect. A low detection limit enables the sensor to capture even very small and subtle changes in force or pressure, making it highly sensitive to delicate touch or low-intensity stimuli. This parameter is particularly critical in applications where precise and accurate detection of minimal forces or pressures is required.

Response time, defined as the interval between the initiation of an external stimulus and the ensuing output response of the sensor, plays a pivotal role in evaluating its performance. The time response parameter requirements of tactile sensors depend on specific application scenarios and needs. In general, in applications that demand quick responsiveness, such as robotics and virtual reality gloves, tactile sensors are expected to exhibit rapid response times, typically in the millisecond range [11,40,41]. For instance, certain piezoelectric tactile sensors can achieve response times as short as nanoseconds, while certain optical-based tactile sensors can achieve response times in the microsecond range [42,43]. The specific response time requirement will depend on the nature of the application and the desired level of precision and real-time feedback. 

In addition to response time, several other important parameters contribute to the overall performance of tactile sensors. One such parameter is stability, representing their ability to maintain consistent and reliable performance over time. A stable sensor exhibits minimal drift or degradation in its sensing capabilities, ensuring long-term accuracy and repeatability. Resolution is another crucial parameter for tactile sensors, which determines the smallest detectable increment of force or pressure that the sensor can accurately perceive. A high-resolution sensor is capable of detecting subtle variations in force or pressure with great precision. The linearity of a sensor is another crucial parameter, indicating how well the sensor’s output follows a linear relationship with the input force or pressure. A highly linear sensor exhibits minimal deviation from linearity, ensuring a reliable and predictable response across a wide range of applied forces or pressures. The dynamic range of a sensor is also significant, referring to the span of input values that the sensor can detect. It represents the difference between the minimum and maximum response values of the sensor. A wide dynamic range enables the sensor to accurately capture both low-intensity and high-intensity stimuli, allowing for versatile and comprehensive sensing capabilities.

## 4. Novel Composites with Functional Polymer

In contrast to the inorganic materials used in conventional tactile sensors, polymer materials possess superior flexibility, lower material density, and reduced manufacturing costs, rendering them more conducive to flexible tactile sensors and contemporary advancements [44]. Therefore, it is necessary to explore new tactile sensor materials. The manifold performance parameters of tactile sensors exhibit a significant correlation with their material selection and structural design. Specifically, the substrate material profoundly impacts the flexibility of the tactile sensor, while the filling material decisively determines the performance parameters such as sensitivity, linear response range, and resolution. In addition, the design process exerts a crucial influence on the overall performance of the sensor. Hence, the investigations of substrate materials, filling materials, structural design, and optimization represents indispensable and pivotal research directions.

The following sections report on different classes of FPCs, including conductive polymer composites, shape memory polymer composites, hydrogels, and liquid crystal elastomers. This discussion focuses on conductive polymer materials for the combination of different fillers to improve their properties. 

### 4.1. Conductive Polymer Composites

Conductive polymer composites (CPCs) are materials that combine a polymer matrix with conductive fillers to achieve a certain level of electrical conductivity [45]. The conductivity of CPCs depends on many factors, such as the type, amount, shape, size, and dispersion of the fillers, as well as the interactions between the fillers and the polymer matrix. The main types of fillers used in CPCs are carbon-based materials, metal-based materials, and conductive polymers.

Carbon nanomaterials: Carbon nanotubes (CNTs) and graphene are widely employed as fillers in CPCs [46]. CNTs, with their unique tubular structure and exceptional electrical properties, form conductive networks within the polymer matrix, thereby significantly improving the electrical conductivity of the composite [47]. Similarly, graphene, a two-dimensional carbon allotrope, offers high conductivity and mechanical strength, making it an excellent candidate for enhancing the properties of CPCs [48]. 

Metal particles: Silver, gold, copper, and nickel metal particles can be incorporated into CPCs by various methods such as in situ synthesis, physical blending, and chemical reduction. These particles provide excellent electrical conductivity and can form interconnected networks within the polymer matrix, facilitating efficient charge transport. Moreover, their high thermal conductivity ensures good heat dissipation in electronic devices.

Conductive polymers: Conducting polymers, such as polyaniline (PANI) [17] and polythiophene, serve as both fillers and functional components in CPCs. These fillers have intrinsic conductivity due to their conjugated π-electron system. They can also exhibit stimuli-responsive properties, such as changing their conductivity under temperature, light, pH, etc. However, they have poor processability, solubility, and environmental stability. These materials possess intrinsic conductivity and excellent compatibility with the polymer matrix, enabling the formation of conductive pathways. These fillers play a crucial role in CPCs by enhancing conductivity, improving mechanical properties, and forming a conductive network.

Advances in polymer functionalization techniques have allowed for the creation of highly specialized tactile sensors with novel characteristics that offer new possibilities for a wide range of applications. The self-healing property of polymers is one of the most extensively studied characteristics, utilized for repairing defects caused by severe deformation during the operation of devices [49,50]. The self-healing properties of CPCs are mainly achieved through two approaches: incorporation of self-healing agents and intrinsic repair. In the first approach, self-healing agents, such as microcapsules or microtubes, are introduced into the conductive material matrix. When the material is damaged, the self-healing agents release repair agents to fill the cracks and restore conductivity. The second approach involves intrinsic repair mechanisms, which leverage the inherent chemical or physical properties of the conductive material itself. This can include mechanisms such as hydrogen bonding, metal coordination bonding, or the thermoplastic nature of the material, which facilitate the automatic closure of cracks and restoration of conductivity. For instance, Sun et al. reported the first comprehensive example of an ionic skin that mimics the features of natural skin, including full-range sensing, self-healing, and strain hardening characteristics [51]. By introducing an entropy-driven supramolecular zwitterionic competition network into a hydrogen-bonded carboxylic acid network, the soft elastic protein network and rigid collagen fibers present in natural skin were simulated, resulting in a unique, mechanically responsive ionic skin.

The self-cleaning characteristic of polymers has garnered considerable attention in recent research, offering the capability to autonomously eliminate pollutants and uphold surface cleanliness, thereby mitigating the adhesion and accumulation of extraneous contaminants [52]. Self-cleaning function can improve the corrosion resistance and pollution resistance of CPCs and reduce their maintenance cost. Self-cleaning functionality in CPCs can be achieved through various mechanisms. One common approach involves surface coating or modification. By introducing specialized coatings or modifiers to the material surface, low surface energy and hydrophobic properties can be attained, making it difficult for contaminants to adhere and enabling self-cleaning effects. This can be achieved through surface coating or the incorporation of photocatalysts such as TiO_2_. Another mechanism involves utilizing the superhydrophobic micro/nanostructures of the material. Controlling the micro/nanostructure can alter the contact angle and surface energy, preventing sufficient contact and the adhesion of pollutants to the surface, thus facilitating self-cleaning effects.

CPCs can achieve biocompatibility by combining them with biodegradable polymers or peptides, enabling tactile sensors and the broad application potential in fields such as tissue engineering and drug delivery. The biocompatibility and biodegradability of conductive polymer materials refer to their ability to be compatible with biological systems, avoid immune rejection or toxic side effects, and their capacity to degrade into harmless substances inside or outside the body [53]. By selecting biodegradable polymers as the matrix, the degradability of tactile sensors can be achieved, reducing their impact on the environment and ecosystems, and enhancing their sustainability and safety.

### 4.2. Shape Memory Polymer Composites

Shape memory polymer composites (SMPCs) are a kind of smart materials based on the shape memory effect, which refers to the ability of a material to recover its original shape after being deformed by an external force, through the external conditions of stimulation such as electricity [54], microwave [55], heat [56], light [57], and chemical induction [58]. Based on their shape memory function, SMPCs can exhibit either a one-way or a two-way shape memory effect. The former refers to the fact that the ability to restore shape is irreversible, while the latter allows the material to reverse its original shape after being deformed and repeated multiple times without losing the original shape memory effect. The memory properties of SMPCs are mainly derived from the dual composition structure of materials: fixed phase and reversible phase, also known as cross-linking points and molecular switches. The fixed phase plays a role in maintaining the original shape of the memory material, which can be chemical cross-linking points (thermosetting) or physical cross-linking points (thermoplastic). The reversible phase can be crystalline or amorphous. It is used to fix the temporary shape of the materials which undergo freezing or softening near their transition temperature, leading to a change in their mechanical properties. Thus, the switching temperature T_trans_ of the SMPs can be either the melting temperature T_m_ or the glass transition temperature T_g_. 

SMPCs are often composed of a polymer matrix reinforced with fillers, such as fiber, CNTs, nanoparticles, organic, and inorganic. The addition of fillers enhances the mechanical properties of the material and also significantly improves the shape memory qualities. In addition to actuation properties such as photothermal responsivity, radiofrequency responsivity and temperature memory effect can also be enhanced.

CNTs filler: CNTs are selected as the reinforcing materials not only because of their superb mechanical, electrical, and thermal properties [59] but also because of their strong microwave absorption [55]. Hence, they can be used to improve the properties of shape memory polymers, such as strength, stiffness, wear resistance, and conductivity [60]. The addition of 50–50 CNT altered the resistivity of the nanocomposite material and manifested 3.82 Ω cm volume resistivity and electro-active shape memory property [61]. Hsu et al. fabricated shape memory polymers incorporated with CNTs [62]. Modified chemical functionalization, improving the dispersion of CNTs in polymer substrates. The shape memory polymer composite containing 4 wt% carbon nanotubes exhibits the highest microwave heating rate, resulting in complete shape recovery within a mere 2 min. 

Organic or inorganic filler: Meppadiyath et al. detailed the implementation of copper-catalyzed azide-alkyne cycloaddition (CuAAC) reaction to achieve crosslinking of triglycidyl isobutyl POSS azide, trisphenyl azide, and propargylated phloroglucinol at their respective cross-linking temperatures, resulting in the synthesis of organic–inorganic hybrid polytriazoles [63]. Notably, the pure organic core-based poly triazole exhibited a glass transition temperature (T_g_) of 35 °C and the hybrid polymers exhibited exceptional shape retention and recovery (>95%) along with thermal stability up to 300 °C.

Overall, the combination of reinforcing fillers in SMPCs can enhance the mechanical properties, thermal stability, shape recovery stress, and stimuli-responsive behaviors of the shape memory polymers, showing potential in biomedical devices, smart textiles, and wearable tactile sensors. 

### 4.3. Hydrogels

Hydrogels are three-dimensional soft materials formed by cross-linked networks of hydrophilic polymer chains, which are capable of swelling and retaining a significant fraction of water. The network structure of hydrogels contains numerous voids that can accommodate a large number of water molecules, allowing the hydrogel to expand [64]. Hydrogels have been exploited as matrix materials for functional composites due to their unique properties including high water content, tunable mechanical properties, biocompatibility, and stimuli responsiveness. 

High water content: Hydrogels possess the remarkable ability to imbibe and retain significant quantities of water, ranging from a mere few percent to an astonishing 90% of their overall weight. The extent of water absorption in hydrogels is contingent upon two critical factors: their cross-linking density and hydrophilicity. Lower cross-linking density coupled with heightened hydrophilicity yields a substantial augmentation in the water absorption capacity of hydrogels.

Tunable mechanical properties: By manipulating the constituent elements of the polymer network, hydrogels possess the ability to finely tune their mechanical characteristics. Factors such as the cross-linking density, polymer chain length, and incorporation of reinforcing agents can be adjusted to tailor hydrogels with precise mechanical properties. Such tailored mechanical properties find significant utility in a multitude of applications, particularly in fields demanding mechanical support, such as the construction of artificial muscles and the development of soft robotics [65].

High biocompatibility: With their innate high biocompatibility, hydrogels demonstrate a remarkable ability to emulate the intricate extracellular matrix found in biological tissues. The integration of hydrogel-based tactile sensors holds vast potential in numerous domains, including wound monitoring, drug delivery systems, tissue engineering, and neural interfacing.

Stimuli responsiveness: Hydrogels can undergo reversible or irreversible changes in their shape, structure, or function in response to external stimuli, such as temperature, pH, electric field, light, magnetic field, etc. This enables hydrogels to achieve smart control and regulation. Temperature-sensitive hydrogels, for instance, contribute to temperature sensing and regulation, while pH-sensitive hydrogels enable pH sensing and regulation.

However, hydrogels face two major challenges: poor mechanical strength and stability due to their uneven network structure, and the issue of dehydration in dry and hot environments. To overcome these challenges, strategies such as organo-hydrogels, ionic hydrogels [66], and CNT hydrogels have been developed. Synthetic polymers such as polyvinyl alcohol (PVA) [67] and polyacrylamide (PAM) [68], biopolymers such as cellulose [69], silk fibroin [70], chitosan [71], and gelatin [72] are commonly used in hydrogel synthesis. Additionally, techniques such as processing and synthesis enable the creation of double-network hydrogels that exhibit improved properties, including enhanced stretchability and toughness [73].

Among them, a noteworthy example of a functional polymer composite hydrogel is the graphene oxide-polyacrylamide hydrogel. For instance, Yu et al. proposed highly stretchable, recoverable, self-healing, and adhesive nanocomposite hydrogels by combining the hybrid core–shell nanoparticles SiO_2_-gPAAm into the PAAc networks [74]. The hybrid nanoparticles acted as physical cross-linkers and stress dissipators in the network, enhancing the mechanical strength and stability of the hydrogel. Their modulus reinforced by in situ-formed polyelectrolyte complex nanoparticles is similar to that of human skin [75], which ranges from 0.1 kPa to 1 MPa depending on the function and location of the tissues. Moreover, this nanocomposite-hydrogel-based sensor showed a high detection sensitivity in the strain range from 50% to 500% with a gauge factor value of 5.86, rapid response time, and good antifatigue performance. 

### 4.4. Liquid Crystal Elastomers

Liquid crystal elastomers (LCEs) are a type of shape memory polymer made up of lightly cross-linked polymer networks that display reversible shape change as they transition from nematic to isotropic phases [76]. LCEs have a highly anisotropic structure due to the presence of both a liquid crystal phase and an elastomer network. The orientation of the liquid crystal molecules within the elastomer matrix can be controlled by external stimuli such as heat, light, or mechanical deformation, resulting in a change in the material’s macroscopic properties.

The LCE composites embedded various fillers including metallic nanoparticles, magnetic particles, liquid metal, CNTs, and graphene derivatives. The coupling between the functionality of fillers and the anisotropic shape deformation from LCEs leads to improving the physical and mechanical properties, such as thermal, optical, and electrical conductivity [77]. The interplay between fillers and the LCEs matrix, as well as techniques for enhancing their compatibility through chemical synthesis and surface modification, are elaborated below.

Liquid metal: The addition of liquid metal to LCEs can significantly improve their mechanical, electrical, and thermal properties. Liquid metal can be incorporated into LCEs with various methods, including infiltration, blending, and coating. The interaction between the liquid metal and LCEs matrix can be improved through chemical synthesis and surface modification strategies [78]. This can enhance the compatibility and adhesion between the two materials, resulting in a more uniform dispersion of the liquid metal within the LCE matrix making them promising materials for various applications such as soft robotics, sensors, and energy harvesting. 

Magnetic particles: The presence of magnetic particles can impart magnetic responsiveness to the LCEs, allowing for remote control and actuation through external magnetic fields. By adjusting the concentration, size, and shape of the magnetic particles, the mechanical properties and response of the LCEs can be tuned to achieve desired outcomes, such as improving actuation speed, sensitivity, and controllability. Additionally, magnetic particles can also enhance the thermal and electrical conductivity of the LCEs, which can be useful for applications in energy harvesting and sensing.

One of the most significant features of LCEs is their reversible shape change in response to external stimuli. For example, rod-shaped LCEs can change their shape into a spiral or helical shape upon heating, and then revert to its original shape upon cooling. This unique shape-shifting behavior is due to the alignment of the liquid crystal molecules within the elastomer matrix, which allows the material to deform in a controlled manner. LCEs also exhibit other interesting properties such as anisotropic thermal expansion, photo-responsive behavior, and optical properties. For example, the optical property of LCEs can be tuned by controlling the orientation of the liquid crystal molecules within the elastomer matrix, allowing for the development of smart optical devices.

In terms of fabrication, LCEs can be synthesized via the incorporation of liquid crystal molecules into a crosslinked elastomer network. The liquid crystal molecules can be either mesogenic (rod-like) or discotic (disk-shaped), and the elastomer can be either silicone-based or polyurethane-based. The synthesis process typically involves a series of steps, including the mixing of liquid crystal and elastomer precursors, crosslinking, and alignment of the liquid crystal molecules through various external stimuli.

## 5. Type of FPCs-Based Tactile Sensors

### 5.1. Piezoresistive Type 

The field of piezoresistive sensors has garnered significant attention with a particular focus on the pressure or strain response exhibited by various FPCs. This mechanism, characterized by its uncomplicated structure, remarkable resolution, chemical stability, and multifaceted advantages, has obtained extensive scrutiny and has been widely applied across various domains. The performance of piezoresistive sensors is usually related to the resistance coefficient of the material, the dispersion of the filling, and structure design. However, the cross-talk between sensors, the inhomogeneity of the filling material, the percolation threshold of functional polymer composites, and the complex manufacturing process significantly hinder their implementation in high-precision applications. Thus, recent research efforts were oriented toward utilizing polymeric composite materials, conductive filler particles, and microstructures to overcome the limitations.

Furthermore, inspired by human skin and the nervous system, bionic piezoresistive tactile sensors developed by simulating human tactile sensing mechanisms have become a research hotspot in recent years [44,79,80,81,82]. Sengupta et al. developed piezoresistive sensors using a bioinspired approach that involved a two-pronged strategy, which utilized carbon nanofiber–polydimethylsiloxane composites in conjunction with spiking neural networks [79], encoding analog voltages to spikes successfully to achieve simulated skin tactile perception. Another example used a carbon-nanofiber-based piezoresistive sensor as a tactile perception receptor, proposed by Chen et al. [83]. Through the ion migration effect to achieve voltage-controlled bipolar resistance switching and multistage resistance memory and the utilization of CsPbBr_3_ digital–analog dual memristors as artificial synapses, the piezoresistive tactile sensor demonstrated a sensitivity of 22.4 kPa^−1^, durability of 1.5 × 10^4^ cycles, and a fast response time of 2.43 ms. 

**Figure 2:**
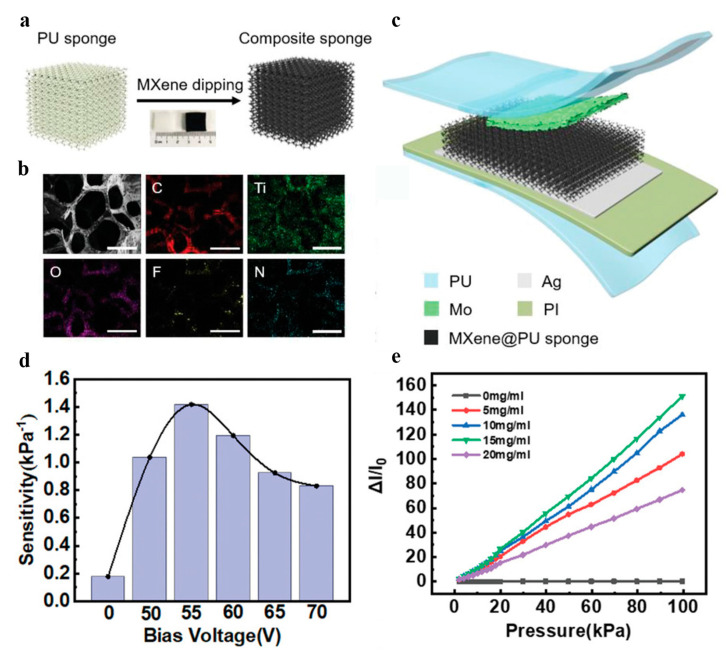
Schematic of piezoresistive tactile sensor. (**a**) Dip-coating method for fabricating MXene-decorated PU sponge. (**b**) C, Ti, O, F, and N for the MXene-coated sponge. (**c**) Schematic illustration of the structure. (**d**) Histogram of linear sensitivity versus voltage. (**e**) The current variation rate (ΔI/I_0_) curves are plotted against pressure for different concentrations of MXene. Reprinted/adapted with permission from Ref. [84]. Copyright 2021, John Wiley and Sons.

For improving the interface coupling efficiency, Wei et al. proposed a flexible piezoresistive sensor based on the MXene@PU sponge [84]. The composite sponge structure was made by dipping a PU sponge into an MXene solution. The MXene nanosheets can adhere to the surface of the PU sponge through physical adsorption and hydrogen bonding. As shown in Figure 2a, after dipping the PU sponge into the MXene solution, the color of the PU sponge changed from white to black due to the optical absorption of the MXene nanoparticle. The energy-dispersive spectrometer result for the MXene sponge structure is depicted in Figure 2c, where can be seen that MXene was evenly distributed in the PU sponge. The resulting MXene sponge composite was then sandwiched between two flexible electrodes (Mo and Ag) with a polyimide film to form the piezoresistive sensor, as shown in Figure 2b. The presented sensing device exhibited exceptional attributes, including a high sensitivity of 1.52 kPa^−1^ and repeatability, alongside remarkable linearity within a broad sensing range of 0–100 kPa (Figure 2d,e). Notably, it achieved a rapid response time of 226 ms for pressure detection, revealing great potential in wearable electronics. 

### 5.2. Capacitive Type 

**Figure 3:**
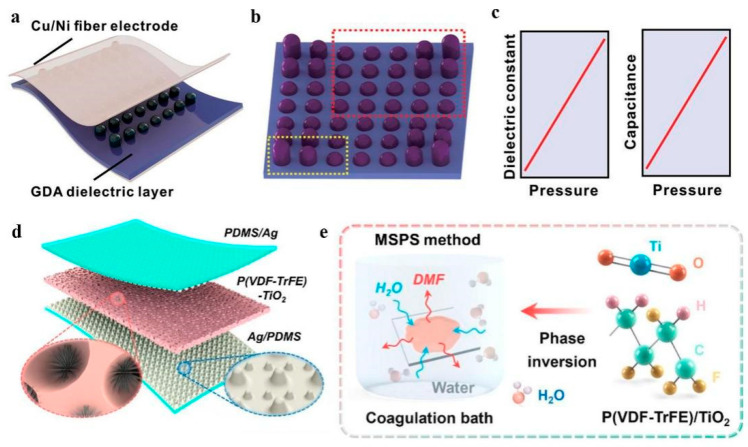
Schematic of capacitive tactile sensors (**a**) Structure of the sensor and (**b**) The gradient-architecture-enabled (GDA) dielectric layer. (**c**) Dielectric behavior of the broad linear sensing. (**d**) Structure of the sensor with heterostructured active layers. Reprinted/adapted with permission from Ref. [87]. Copyright 2021, John Wiley and Sons. (**e**) The MSPS process: a mixture of solvents is employed to create a controlled phase separation, which leads to the formation of a structured hierarchical sea urchin TiO_2_ particle-in-micropore morphology. Reprinted/adapted with permission from Ref. [23]. Copyright 2022, Elsevier.

Capacitive tactile sensors have garnered significant attention in the fields of soft robotics, human–machine interactions, medical devices, and wearable technology due to their remarkable advantages such as low power consumption, rapid response time, and simple device structure. Notably, extensive research has been conducted to augment the sensitivity and linear response of capacitive sensors based on FPCs for an extensive range of pressure detection scenarios. Material selection plays a crucial role in determining the properties of the dielectric layer. Polymers, elastomeric composites, and other flexible materials are often preferred choices due to their desirable characteristics such as processability, deformability, lightweight nature, and chemical resistance. To date, polydimethylsiloxane (PDMS), which exhibits a low Young’s modulus (≈1.26~1.72 MPa) [85], has emerged as the predominant choice. PDMS exhibits both viscous and elastic behavior, which can lead to slower response and recovery times compared to more rigid or less viscoelastic materials. To mitigate the prolonged response and recovery time associated with PDMS, researchers are exploring various strategies. One approach involves the integration of highly sensitive nanomaterials, such as carbon nanotubes (CNT) or graphene, into the capacitive sensing structure. For instance, Fu et al. introduced a flexible capacitive tactile sensor that utilized a layered structure consisting of CNT/ PDMS film sandwiched between two polyimide films with patterned electrodes and a scaffold architecture composed of parylene films [85]. This innovative design exhibited outstanding performance, including a high sensitivity of 1.61% kPa^−1^ for pressures below 1 MPa and a wide pressure working range spanning from 0.9 kPa to 2.55 MPa. Furthermore, microstructures or nanostructures between the electrode plates, as well as multilayer or multichannel structures, have been used to optimize the capacitive characteristics of devices. Zhang et al. designed a planar four-capacitor capacitive tactile sensor by incorporating stopper structures, gaining high sensitivity (0.901 pF/N) under a normal force and wider dynamic range [86]. A novel capacitive tactile sensor utilizing a gradient architecture was introduced by Ji. al [87]. The design involved the integration of a structured gradient insulating layer placed between two facing Cu/Ni fiber electrodes, along with a gradient conductive and spacer layer, as illustrated in Figure 3a,b. This configuration allowed for enhancing sensitivity and performance. Through systematic optimization techniques, the sensor demonstrated a sensitivity of 0.065 kPa^−1^, exhibiting excellent linearity characteristics as depicted in Figure 3c. Notably, the linearity range extended up to an impressive 1700 kPa, showcasing its potential for accurately detecting a wide range of applied pressures. 

However, achieving a clear and stable perception of tiny signals can pose a significant challenge. Factors such as noise, interference, and signal attenuation can hinder the reliability and precision of signal perception, particularly when dealing with minuscule signals. Niu et al. introduced a novel approach for the design and fabrication of capacitive tactile sensors, employing dielectric and electrode layers with sparsely spaced microstructures [23]. As indicated in Figure 3d, the dielectric layer was composed of PVDF-TrFE-TiO_2_, featuring a hierarchical sea urchin TiO_2_ particle-in-micropore structure and the electrode layers consisted of AgPDMS. The fabrication process, illustrated in Figure 3e, involved the mixed solvent phase separation method. The sensor demonstrated remarkable characteristics, including pressure sensitivity of 10.5 kPa^−1^ below 100 Pa, fast response and relaxation times both of 5.6 ms, an ultralow limit of detection down to 0.1 Pa, as well as excellent compression and bending stability withstanding up to 10,000 and 12,000 cycles, respectively. 

When it comes to electrodes, key considerations include the need for exceptional stability, excellent conductivity across a wide range of strains, and exceptional flexibility. Techniques for fabricating flexible electrodes include printing, deposition, and solution processing [87]. Elastomeric composites that incorporate conductive materials show great promise as flexible electrodes. Moreover, the electrode pattern or design can impact the sensor’s performance. Various electrode patterns, such as interdigitated [88,89] or parallel plate configurations [90], can be employed to optimize the sensing capabilities. The pattern should be carefully designed to ensure sufficient coverage, minimize fringing effects, and maximize the changes in capacitance in response to applied force or pressure.

### 5.3. Piezoelectric Type

Piezoelectric sensors, coupled with the advancements in piezoelectric nanogenerator (PENG) technology, offer an impressive array of benefits. With their extensive dynamic range, exceptional sensitivity, minimal power consumption, and rapid response time, these sensors are capable of accurately capturing and converting mechanical stimuli into electrical energy. This dual functionality not only enables precise force and pressure measurements, but also harnesses the generated electricity for various applications, such as powering small electronic devices or contributing to energy harvesting systems. The efficacy and functionality of PENG tactile sensors are intricately linked to the inherent properties, material compositions, and performance of the employed piezoelectric materials. As the key components of PENG, these piezoelectric materials play a pivotal role in converting mechanical stimuli into electrical energy, facilitating the efficient generation of power. The selections of suitable piezoelectric materials with desirable properties, such as high piezoelectric coefficients, excellent mechanical durability, and low electrical leakage, are critical in optimizing the performance and overall effectiveness of PENG-based sensors and energy harvesting devices. Traditional piezoelectric materials such as quartz, crystalline, or ceramic possess excellent piezoelectric properties, high sensitivity, and stability, but drawbacks high brittleness, low flexibility, and limited processability difficult to meet the high sensing requirements. Therefore, extensive research and development efforts are dedicated to exploring and engineering advanced piezoelectric materials to enhance the performance and broaden the application potential of PENG systems.

One approach to improve the properties of piezoelectric materials is to fabricate polymer–inorganic composites that combine the advantages of both components. Commonly used inorganic piezoelectric materials include lead-zirconium titanate (PZT) [91], ZnO [92], and barium titanate (BaTiO_3_) [93]. Due to the inherent brittleness of inorganic piezoelectric materials, they are prone to fracture or breakage under mechanical stress. To overcome this limitation, these inorganic materials can be integrated into a polymer matrix in diverse forms, such as nanoparticles, nanowires, nanorods, and nanosheets. The polymer matrix can provide flexibility and versatility in terms of design and manufacturing, as well as enhance the mechanical strength and durability of the composites. Organic piezoelectric materials, including PVDF and its copolymers [93,94,95,96,97,98], PVDF-TrFE [94,98], and PLA [99] are another class of promising materials for PENG systems. These materials have lower piezoelectric and electromechanical properties than inorganic ones, but they exhibit a range of advantageous properties, such as being biocompatible, lightweight, easy to process, and relatively cheap.

In the pursuit of developing highly efficient and versatile piezoelectric sensors, researchers have turned their attentions toward hybrid materials that combine organic and inorganic components. The hybridization of organic polymers with specific mechanical and electrical properties and inorganic components with inherent piezoelectric properties can synergistically enhance the overall piezoelectric response and performance of the materials. For instance, M et al. incorporated ZNO, BaTiO_3_, and other inorganic nanoparticles into PVDF matrix device resulting in a significant increase in the β-phase content and enhanced piezoelectric properties [97]. PVDF-ZnO-based, PVDF-TiO_2_-based, and PVDF-SiO_2_-based sensors were observed with a sensitivity of 103 mV/N, 85.6 mV/N, and 60.7 mV/N, respectively. 

**Figure 4:**
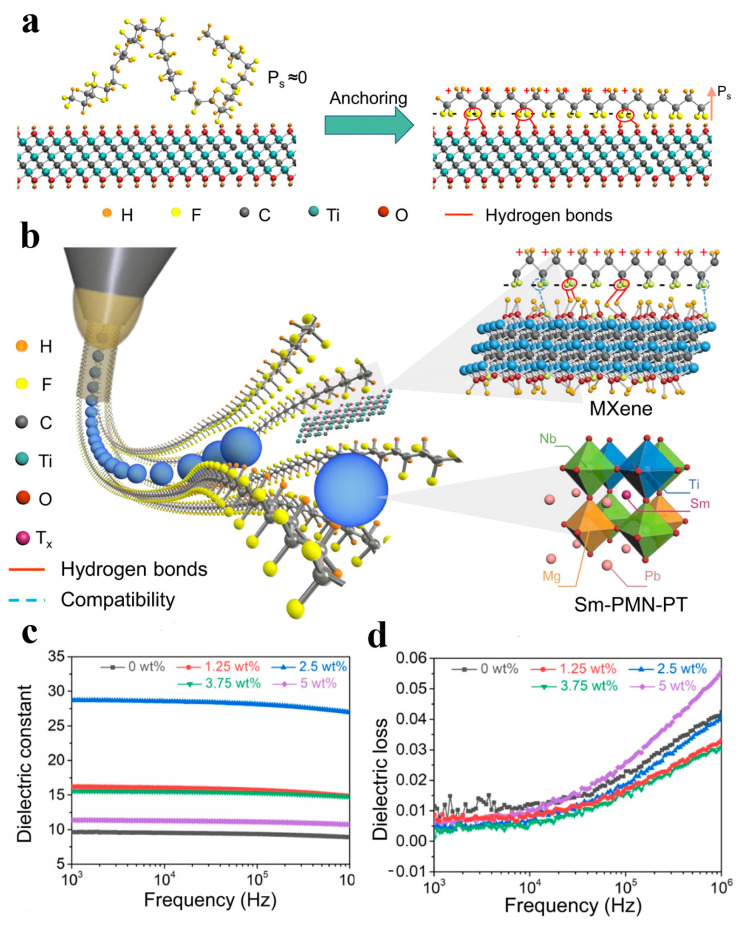
Schematic of the soft piezoelectric textile sensor. (**a**) In situ stretching and alignment of PVDF polymer chain via surface terminations on Ti_3_C_2_T_x_ nanosheets to upgrade the spontaneous polarization. (**b**) The structure design of piezoelectric composite and electrospinning procedure. (**c**) The dielectric permittivity dependent on frequency. (**d**) The dielectric loss dependent on frequency. Reprinted/adapted with permission from Ref. [100]. Copyright 2022, Springer Nature.

Furthermore, nanofiber technology has attracted considerable attention for the fabrication of conductive nanofiber networks with high surface-to-volume ratios and large porosity. Electrospinning is a versatile and widely adopted technique that involves the use of an electric field to create ultrafine fibers from a polymer solution or melt. One example of applying electrospinning to design flexible piezoelectric tactile sensors was proposed by Chen et al. [96]. PVDF-based nanofibers were synthesized with Fe_3_O_4_ nanoparticles and formed into a cilia-inspired structure on a substrate, which could promote the formation of β-phase in PVDF and enhance its piezoelectric properties. It demonstrated an impressive output voltage of 4.52 V and a stable performance over 30,000 cycles of vibration testing. Another example of using electrospinning to fabricate high-performance piezoelectric composites is the work by Su et al. [100], who integrated MXene, samarium-doped Pb (Mg_1/3_Nb_2/3_) O_3_-PbTiO_3_ (Sm-PMN-PT), and PVDF into a soft piezoelectric textile. Figure 4a,b individually show the obtained nanofibers with in situ stretching and local poling and the structure and component of the piezoelectric composites. The addition of MXene and Sm-PMN-PT improved the conductivity and piezoelectricity of the composites. The dielectric permittivity of the composites reached its peak value at 2.5 wt% MXene over a frequency range of 1 kHz to 1 MHz (Figure 4c), while the dielectric loss related to energy dissipation reached its maximum value at 5 wt% MXene (Figure 4d). The optimal MXene concentration improved the interfacial polarization, while excessive MXene addition led to increased leakage current and higher dielectric loss.

Various strategies have been employed to improve the properties of piezoelectric materials, such as forming polymer composites, hybridizing organic–inorganic components, and fabricating nanofiber networks [101]. The selection of suitable piezoelectric materials is crucial for achieving high-performance PENG sensors. However, there are still some challenges and limitations that need to be addressed for further development of PENG systems. Optimizing the design and fabrication of PENG devices for multifunctionality, miniaturization, and integration calls for more interdisciplinary research efforts from different fields of science and engineering.

### 5.4. Triboelectric Type

**Figure 5:**
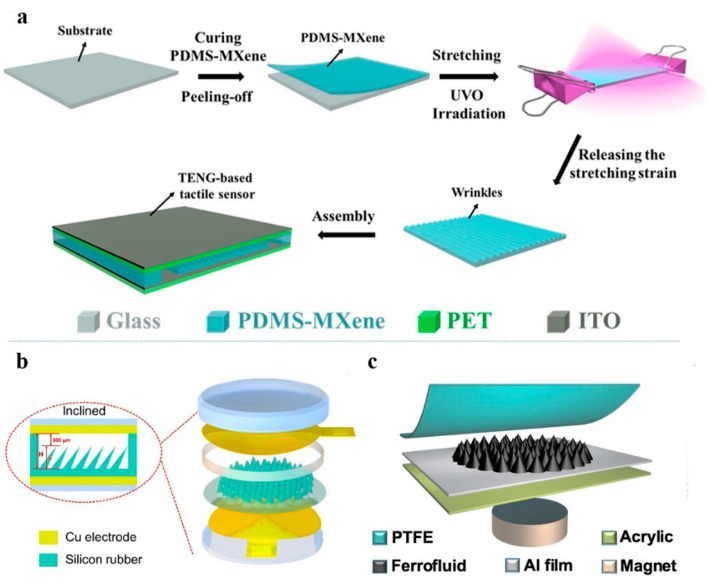
(**a**) The manufacturing procedure of TENG-based tactile sensor. Reprinted/adapted with permission from Ref. [32]. Copyright 2021, Elsevier. (**b**) A sectional view and 3D structure figure of the tactile sensor featuring inclined microneedles. Reprinted/adapted with permission from Ref. [106]. Copyright 2021, Elsevier. (**c**) The basic structure of the triboelectric tactile sensor. Reprinted/adapted with permission from Ref. [107]. Copyright 2022, Springer Nature.

In contrast to piezoelectric sensors that necessitate specific materials possessing inherent piezoelectric properties, such as ceramics, crystals, and polymers, triboelectric sensors offer the advantage of utilizing a diverse array of materials exhibiting distinct triboelectric polarities, such as polymers, metals, fabrics, etc. In addition, the working mode of the TENG affects the selection and performance of the materials for the triboelectric sensors. Four fundamental working modes exist: lateral sliding mode, single-electrode mode, vertical contact separation mode, and freestanding triboelectric layer mode [102,103]. Among these, the single-electrode mode of the TENG stands out due to its simple configuration and easy fabrication. The simplicity of its operation, requiring only the connection of the electrode to a triboelectric surface, surpasses that of paired electrodes TENG [104]. In the pursuit of enhancing the sensing capabilities of triboelectric sensors, significant efforts have been dedicated to their performance. An alternative approach to enhance sensitivity involves the doping of a polymer matrix with dielectric or conductive materials, such as graphite [44,105], silver nanoparticles, carbon nanotubes, and graphene. These materials can increase the surface charge density and conductivity of the polymer matrix, thus improving the triboelectric effect. For example, Chen et al. developed a 3D printing method to prepare stretchable elastic fibers with a coaxial core-sheath structure for self-powered e-skin. The conductive core is made of PDMS mixed with graphene, and the insulative sheath is made of PDMS mixed with PTFE particles. By incorporating conductive and insulating elements at the fiber level, the device structure can be streamlined, leading to compact dimensions and enhanced resolution in tactile sensing. Another example is proposed by Cai et al., who fabricated a highly sensitive triboelectric tactile sensor based on wrinkled PDMS doped with MXene [32]. The sensor demonstrated exceptional sensitivity, particularly when subjected to pressures under 800 Pa. As shown in Figure 5a, the PDMS/MXene composite films were prepared by mixing PDMS prepolymer with MXene nanosheets and then curing and stretching them under ultraviolet ozone (UVO) irradiation. The UVO irradiation induced surface oxidation and wrinkling of the PDMS/MXene composite films, which increased their surface charge density and conductivity. 

One of the key factors that affect the performance of triboelectric tactile sensors is the surface microstructure of the active layer. The surface microstructure can increase the contact area and friction force between the triboelectric layers, thus enhancing charge generation and transfer. However, most of the existing methods to fabricate surface microstructures, such as photolithography, laser etching, and 3D printing, are complicated, costly, and difficult to control dynamically. Therefore, a novel and facile approach based on ferrofluid has been proposed to achieve surface microengineering of the active layer. Ferrofluid is a colloidal suspension of magnetic nanoparticles dispersed in a carrier fluid with a surfactant. Ferrofluid has both the fluidity of a liquid and the magnetism of a solid, which enables it to form various shapes and patterns under an external magnetic field. Zhang et al. used ferrofluid as a template to fabricate micro-spikes on PDMS films as triboelectric layers [106]. The sensor design employed a two-step reversal method, which consisted of a protective layer made of silicon rubber and an electrode made of copper film, as illustrated in Figure 5b. By micro-engineering the dielectric surface of the tactile sensor with varying inclination angles and lengths, it was observed that smaller inclination angles and greater heights positively influenced the sensitivity and expanded the high sensitivity detection range. Liu et al. used ferrofluid itself directly as a liquid triboelectric layer [107]. As illustrated in Figure 5c, the sensor had a single-electrode structure with an aluminum film as the electrostatic induction electrode and a PTFE film as the negative triboelectric layer. The ferrofluid was injected into the aluminum foil as the positive friction layer, and PTFE film was used as the negative layer. A permanent magnet was used to provide the external magnetic field. When a magnet was placed, the ferrofluid immediately formed many spike structures under the influence of an external magnetic field. The sensor demonstrated an impressively high sensitivity of 21.48 kPa^−1^ within the pressure below 2.5 kPa. 

## 6. Applications of Tactile Sensing

### 6.1. Multifunctional Electronic Skin

Human skin is an exceptional organ comprising an integrated and stretchable network of sensors that relay information about thermal and tactile stimuli to the brain. This sensory network detects an array of characteristics, such as shapes, sizes, textures, and levels of contact pressure, allowing humans to maneuver safely and efficiently in their surroundings. Inspired by the sensory capabilities of human skin and leveraging advancements in artificial intelligence, materials science, and other technologies, researchers have made significant strides in the development of e-skin with human-like sensory abilities. E-skin can not only mimic the natural skin in certain aspects, but also integrate additional functionalities and required properties that surpass the natural skin. For instance, Rao et al. developed a tactile e-skin based on a single-electrode-mode TENG made of reduced graphene oxide and Bi_4_Ti_3_O_12_ [108]. The e-skin not only possesses the ability to discriminate between temperature and pressure but also exhibits excellent flexibility. It can simultaneously detect and distinguish between temperature and pressure in real time with high sensitivity and a fast response time.

**Figure 6:**
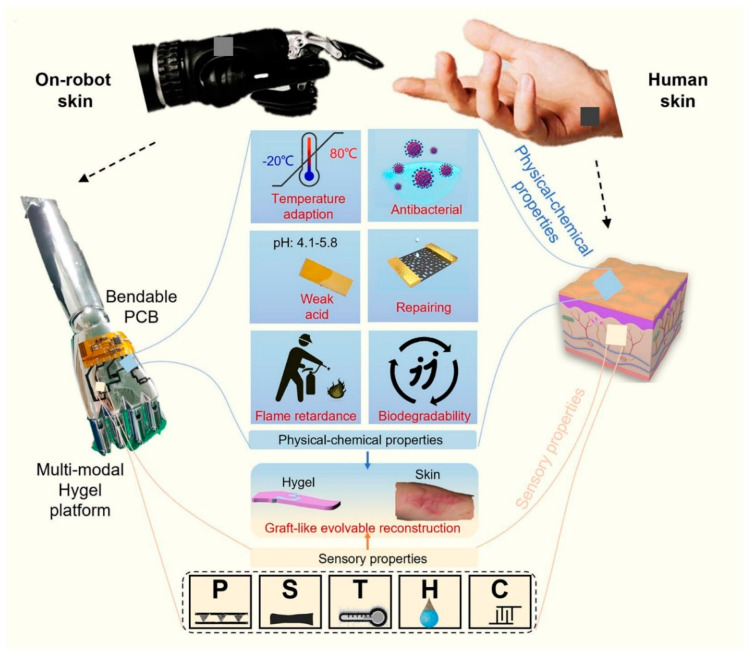
Hygel e-skin covers all the hyper-attributes (physical–chemical and sensory properties) of the human skin, including weak acidity (pH: 4.1–5.8), injury repair, biodegradability, temperature adaptivity, and versatile sensory capabilities via various mechanoreceptors (cold and hot receptors and pain receptors). Reprinted/adapted with permission from Ref. [109]. Copyright 2023, American Chemical Society.

One of the major challenges in e-skin research is to achieve a self-healing function that can restore the mechanical and electrical properties of the damaged e-skin. Although some self-healing mechanisms have been developed, improvements are needed to ensure the long-term functionality and durability of e-skin under various environmental conditions. Duan et al. developed a water-modulated biomimetic hyper-attribute-gel e-skin (Hygel e-skin) that mimics the physical–chemical and sensory properties of human skin [109]. The Hygel e-skin possesses various skin-like attributes such as stretchability, self-healing, biocompatibility, biodegradability, weak acidity, antibacterial activities, flame retardance, and temperature adaptivity. It is capable of detecting and measuring pressure, temperature, humidity, strain, and contact with high resolution and accuracy. Moreover, it can also undergo reversible gel–solid transition via water or sweat modulation, enabling function reconfigurability and evolvability. As depicted in Figure 6, the Hygel e-skin was attached to a robot to demonstrate its highly skin-like attributes in capturing multiple pieces of sensory information and reconfiguring required features, and its excellent skin compatibility for real-time gesture recognition through deep learning. Achieving a biomimetic e-skin that covers all the physicochemical and sensory properties of human skin, leading to more complex and versatile biomimetic applications, is the next-generation goal of artificial skin [110,111]. 

### 6.2. Human–Machine Interaction

In recent years, human–machine interaction (HMI) has emerged as a promising field thanks to the rapid development of computer science, artificial intelligence, 5G communication, and biomimetic materials [112]. The traditional interaction paradigm based on the graphical interface has gradually evolved to incorporate multichannel input information and multimodal information expression. Various types of input information, such as vision, hearing, touch, language, expression, eye movement, gesture, and posture, are being utilized to interact with computer systems and enhance the user experience. Numerous studies have been conducted on the applications of HMI, with a particular focus on areas such as smart wireless control, intelligent keyboards, and robots with active perception.

**Figure 7:**
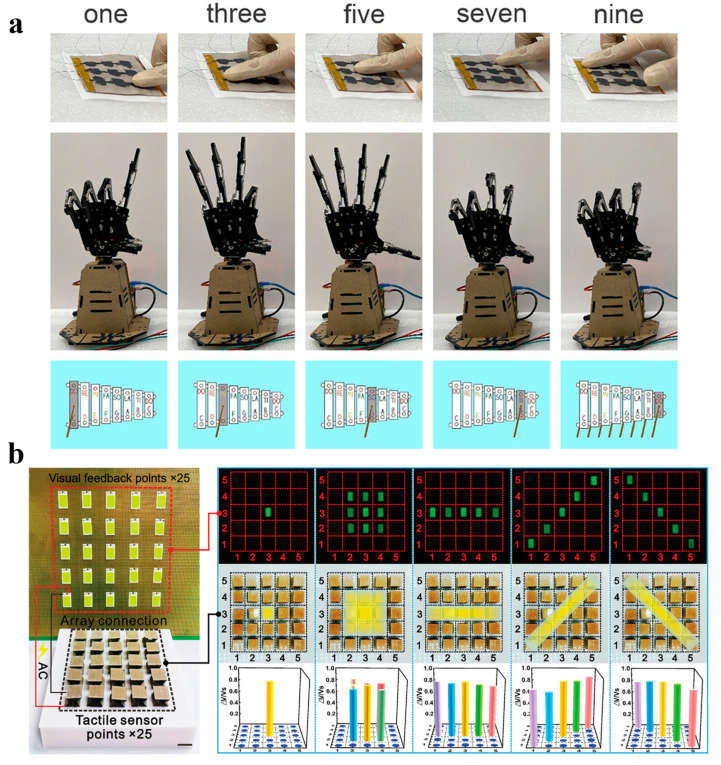
The HMI Applications in Tactile Sensor. (**a**) Demonstrated by the corresponding actions made by the mechanical hand and the controlled music keyboard after pressing the array. Reprinted/adapted with permission from Ref. [113]. Copyright 2023, American Chemical Society. (**b**) An active matrix (5 × 5) design strategy was used to develop traceable visual tactile sensing. The scale bar is 2 cm. The tactile sensors generated visualized light distribution mapping and electrical signal distribution maps at different locations. Reprinted/adapted with permission from Ref. [41]. Copyright 2022, John Wiley and Sons.

Zhong et al. proposed a flexible tactile sensor array based on the textile woven structure by setting finger tapings and sounds to be signals as well as feedback, which can achieve a wearable HMI device similar to an electronic drum [40]. This device can realize single-point, multipoint, and sliding touch functions, which can be used for various HMI applications such as somatosensory games, intelligent robot systems, and artificial intelligence. Tao et al. developed a tactile hydrogel sensor with remarkable self-powered sensing capabilities, which functions as a switching button for the control of electrical appliances and robotic hands, mimicking human finger gestures [73]. Zhang et al. introduced a 3 × 3 cross-interactive tactile sensor array (Figure 7a), which was successfully applied to effectively manipulate mechanical hand movements and keyboards in computer programs to play music [113]. The sensor array can achieve high output voltage and power density under low-pressure stimuli. Another example of a capacitive magnetic field/pressure sensor was proposed by Li et al., which can recognize various braille characters in a contactless manner through a flexible arrangement of permanent magnets (Figure 7b) [41]. Notably, the sensor was also proposed to realize visual sensing of the palm grasp state and strength feedback without an external power supply.

### 6.3. Health and Sports Monitoring

**Figure 8:**
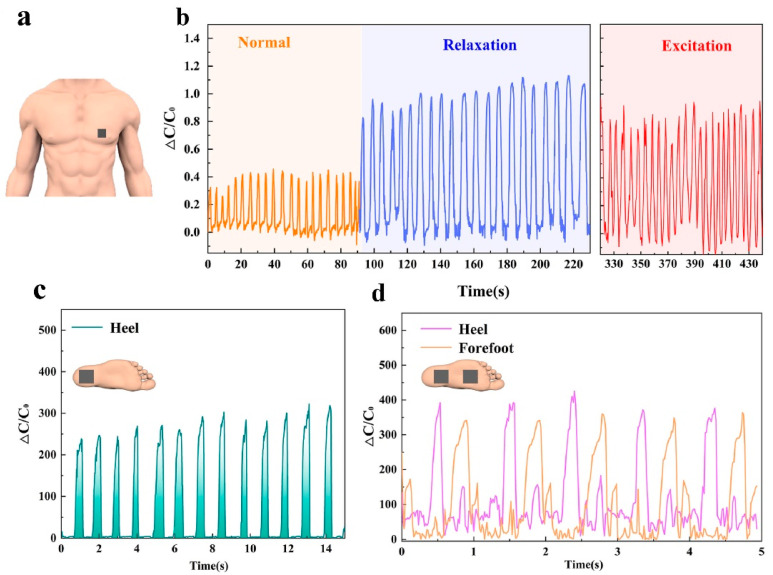
Detection of various physical movements and exerted pressure. (**a**) Schematic illustration of the flexible sensor attached to the human chest for breathing detection. (**b**) The attachment of a flexible sensor to the human chest facilitates the detection of breathing patterns. (**c**) The sensor for plantar pressure detection during walking. (**d**) Capacitance changes of heel and forefoot sensors during gait monitoring. Reprinted/adapted with permission from Ref. [115]. Copyright 2021, Elsevier.

**Figure 9:**
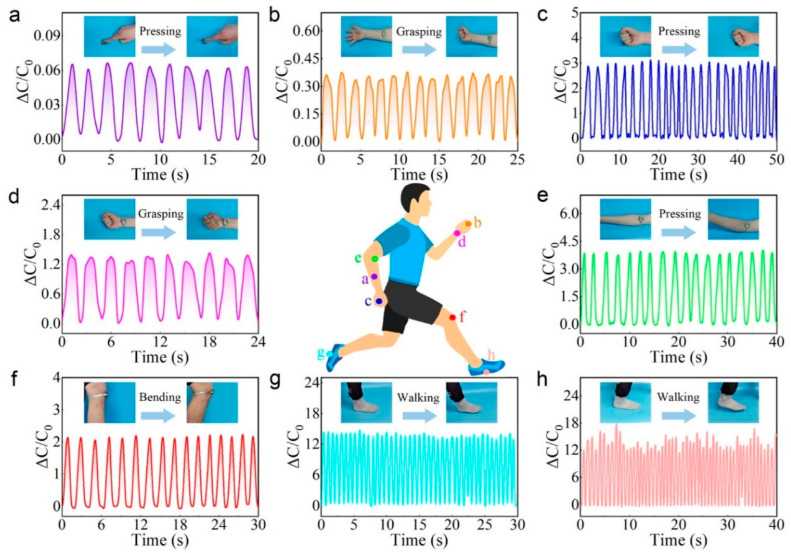
Human motion detection: (**a**) finger touch, (**b**) arm muscle movement, (**c**) clenched fists touching, (**d**) wrist bend, (**e**) elbow flexion, (**f**) knee flexion, (**g**) balance walking, and (**h**) unbalanced walking. Reprinted/adapted with permission from Ref. [116]. Copyright 2022, American Chemical Society.

Tactile sensors have evolved beyond their traditional role of mimicking human skin and sensing external stimuli, which possess unique capabilities that surpass those of human skin, enabling them to monitor vital signs and contribute to personal health protection systems. By detecting and analyzing signals related to human blood pressure, heartbeat, and breathing rate, tactile sensors can identify and alert users or medical professionals about abnormal health conditions [114]. By utilizing these advanced capabilities, tactile sensors contribute to the development of personalized healthcare systems that empower individuals to monitor their health in real time. These sensors provide valuable data for the early detection and prevention of health issues, allowing for timely intervention and treatment. Furthermore, they can assist medical professionals in remote patient monitoring, providing patients’ vital signs. Chen et al. fabricated a capacitive tactile sensor that adopts a biomimetic hierarchical array architecture coated with silver nanowires [115]. This innovative sensor design enabled the real-time detection of large amounts of pressure, making it highly suitable for applications in healthcare monitoring and body motion recognition. The sensor can discern and differentiate subtle arterial pulse signals under various age, gender and motion conditions, as well as monitor the physiological activities under high pressure such as respiration (Figure 8a,b) and plantar pressure (Figure 8c,d). Additionally, flexible bionic tactile sensors with high sensitivity and fast response based on the octopus suction cup microstructure were proposed [116]. The novel structure can endow the sensor with excellent sensing ability, making it an ideal capacitive sensor for the high-precision detection of objects grasped by bionic manipulators and wearable devices for monitoring human motion posture. The illustration in Figure 9 demonstrates how the sensors are attached to different parts of the user’s body to detect and monitor various types of body movements.

## 7. Conclusions

In this review, we highlighted the main progress in the development of FPCs-based tactile sensors and their applications in e-skin, human–machine interaction, and health monitoring. The booming development of new materials and novel fabrication methods leads to continuous advances in sensor performance. FPCs offer many advantages for tactile sensor applications, such as excellent processability, inherent ductility, biocompatibility, biodegradability, and multifunctionality. Table 1 presents a comparison of different transduction mechanisms employed in tactile sensors, focusing on their sensitivity, response time, and cycle durability. By combining FPCs with different transduction principles, sensor structures, and fabrication methods, researchers have achieved remarkable achievements in enhancing the functionality and adaptability of tactile sensors. Some unique and desired properties such as self-healing, self-powering, visualization, and multisensory integration have already been realized by using FPCs. Conductive polymers are a noteworthy class of FPCs that have been widely used for tactile sensors due to their high conductivity and flexibility. The combination of these materials with elastomer substrates holds immense potential for developing advanced tactile sensing devices with enhanced functionality and adaptability.

However, there are still some challenges and limitations that need to be overcome before FPCs-based tactile sensors can be widely applied in real-world scenarios. The trade-off between the mechanical and electrical properties of FPCs directly impacts the selection of filler concentration and the optimization of composite morphology. To achieve an optimal balance between device deformability and charge transport capability, hierarchical structures or hybrid materials are often employed. Furthermore, the signal processing and transmission of FPCs-based tactile sensors may encounter some issues such as signal noise, interference, attenuation, and power consumption. To address these issues, advanced technologies such as wireless communication, energy harvesting, and neuromorphic computing can be integrated with FPCs-based tactile sensors to enable more intelligent and autonomous sensing systems.

These challenges provide opportunities for further research and development of FPCMs-based tactile sensors. The ultimate goal is to achieve a biomimetic tactile sensor that can fully mimic and even surpass natural skin in terms of functionality and performance.

Future research directions in the field of tactile sensors include:

1. Miniaturization and integration: investigating methods to miniaturize tactile sensors and integrate them into compact and portable devices, enabling their widespread use in various applications, including wearable electronics, robotics, healthcare, and virtual reality.
2. Multimodal sensing: integrating multiple sensing modalities, such as pressure, temperature, vibration, and texture, into a single tactile sensor to provide comprehensive and rich haptic feedback, mimicking the human sense of touch more accurately; in addition, establishing effective neural interfaces that can connect functional polymer composite tactile sensors with the human nervous system to achieve bidirectional information transfer and feedback.
3. Energy harvesting: exploring the incorporation of energy harvesting capabilities into tactile sensors to enable self-powering or energy-efficient operation, thereby reducing reliance on external power sources and expanding their potential applications.
4. Artificial intelligence (AI) and machine learning (ML): taking advantage of AI and ML algorithms to enhance the data processing and interpretation capabilities of tactile sensors, enabling more advanced and intelligent analysis of touch.
5. The development of advanced materials: exploring new materials and composites with enhanced mechanical and electrical properties, such as self-healing materials, stretchable conductors, and multifunctional nanomaterials, to further improve the performance and durability of tactile sensors.

## Figures and Tables

**Table 1:** Sensing performance of different types of state-of-the-art tactile sensors.

Year	Sensing Principle	Materials	Sensitivity/Sensing Range	Low Detection Limit (Pa)	Response Time/Recovery Time (ms)	Stability (Cycle)	Ref.
2021	Piezoresistive	MWCNTs/PDMS	6.821 kPa^−1^/10–20 kPa 0.029 kPa^−1^/30–200 kPa	-	1.6/-	10,200	[15]
2022	Piezoresistive	MXene@PU-sponge	1.47 kPa^−1^	-	323/226	6000	[84]
2023	Piezoresistive	SWCNTs/PDMS	1.28 × 10^6^ kPa^−1^	0.10	149/138	2000	[117]
2020	capacitive	PVDF-TrFE	6.583 kPa^−1^ /0–100 Pa	3	48/36	10,000	[24]
2021	capacitive	CNT/PDMS	1.61% kPa^−1^/<1 MPa	-	60/45	5000	[85]
2022	Capacitive	PVDF-TrFE-TiO_2_	10.5 kPa^−1^ /<100 Pa	0.1	5.6/5.6	12,000	[23]
2023	Capacitive	PDMS/silver ink	0.76 kPa^−1^/<1 kPa	2	34/28	-	[118]
2020	Piezoelectric	PVDF nano arrays	5.17 kPa^−1^	175	150	30,000	[119]
2021	Triboelectric	Wrinkled PDMS/MXene	18 VPa^−1^/10–80 Pa 0.06 VPa^−1^/80–800 Pa	-	-	10,000	[32]
2022	Triboelectric	Pyramid ionic hydrogel/PDMS	45.97 mVPa^−1^	20	17/23	36,000	[73]
2022	Triboelectric	Ferrofluid/PTFE	21.48 kPa^−1^	1.25	90/-	10,000	[107]
2023	Triboelectric	FEP/chitosan	2.93 kPa^−1^	-	51/-	100,000	[41]
2021	Piezoelectric	PVDF/Fe_3_O_4_	-	-	-	30,000	[96]
2023	Piezoelectric	PVDF/ZnO	103 mV/N	-	-	200	[97]
2023	Piezoelectric	PVDF/SiO_2_	60.7 mV/N	-	-	200	[97]
2023	Piezoelectric	PVDF/TiO_2_	85.6 mV/N		-	200	[97]

CNT: carbon nanotubes; MWCNTs: multiwalled carbon nanotubes; PDMS: polydimethylsiloxane; SWCNTs: single-walled carbon nanotubes; PVDF: poly (vinylidene fluoride); PVDF-TrFE: poly (vinylidene fluoride-trifluoroethylene); FEP: fluorinated ethylene propylene.

## Data Availability

Not applicable.
